# Supplementary material for: Tead1a Initiates Transcriptional Priming Through the TEAD1a/YAP‐Notch1‐Spi1/Cebpα Axis to Promote Neutrophil Fate
Source: Adv Sci (Weinh). 2025 Aug 28;12(41):e05441. doi: 10.1002/advs.202505441 (PMC12591127; doi:10.1002/advs.202505441)
Supplement: Supplementary file 4 — Supplementary Dataset [file ADVS-12-e05441-s004.zip › 3-6.zip!/3-6/02-6.cluster/cluster15/Enrich/ego_BP.pdf]

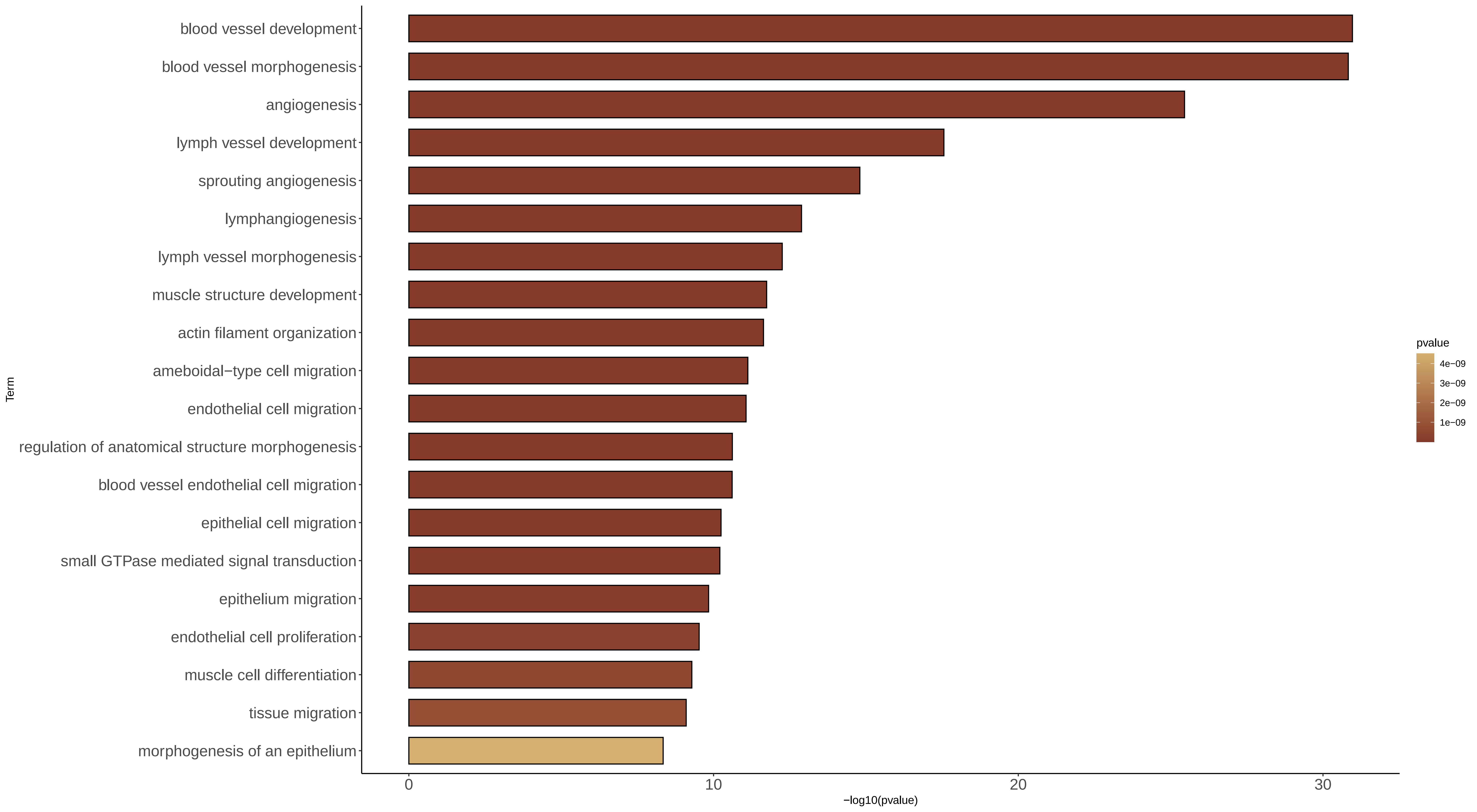

blood vessel development
blood vessel morphogenesis
angiogenesis
lymph vessel development
sprouting angiogenesis
lymphangiogenesis
lymph vessel morphogenesis
muscle structure development
actin filament organization
ameboidal−type cell migration
endothelial cell migration
regulation of anatomical structure morphogenesis
blood vessel endothelial cell migration
epithelial cell migration
small GTPase mediated signal transduction
epithelium migration
endothelial cell proliferation
muscle cell differentiation
tissue migration
morphogenesis of an epithelium
Term
0
10
20
30
−log10(pvalue)
pvalue
4e−09
3e−09
2e−09
1e−09
